# Global Trends in LADA Type Diabetes Research: A Bibliometric Analysis of Publications from Web of Science and Scopus, 1994–2024

**DOI:** 10.1155/2024/4960075

**Published:** 2024-10-14

**Authors:** Khatimya Kudabayeva, Bibigul Tleumagambetova, Yerlan Bazargaliyev, Raikul Kosmuratova, Aliya Zhylkybekova

**Affiliations:** ^1^Department of Internal Diseases 1, West Kazakhstan Marat Ospanov Medical University, Aktobe, Kazakhstan; ^2^Department of Propaedeutics of Internal Disease, West Kazakhstan Marat Ospanov Medical University, Aktobe, Kazakhstan; ^3^Department of Pathophysiology, West Kazakhstan Marat Ospanov Medical University 030012, Aktobe, Kazakhstan

**Keywords:** autoimmune diabetes in adults, bibliometric analysis, latent autoimmune diabetes of adults, noninsulin-requiring autoimmune diabetes, slow progressing insulin-dependent diabetes mellitus, Type 2 diabetes mellitus

## Abstract

The prevalence of T2DM has been increasing dramatically over recent decades, about 537 million people in 2021. LADA type diabetes, a subtype of diabetes that exhibits characteristics of both T2DM and autoimmune beta-cell destruction similar to T1DM, but with a later onset. The aim of this study is to analyze the main research field on LADA type, including analysis of countries, institutions, journals, authors, and keywords. This research utilized a descriptive bibliometric design. We collected and analyzed data from 672 publications indexed in the Web of Science and Scopus databases, covering the period from 1994 to January 2024. The bibliometric analysis included English-language research articles that involved studies on patients with LADA type diabetes, aged 18 years or older. RStudio and the Bibliometrix R package were used for data merging and for performing statistical and visual analyses. The annual publication shows an upward trend over the period, with the highest number of publications per year in 2021. The study showed that China leads in the number of articles, with 101 papers published. The United Kingdom demonstrates significant international collaborations, particularly with Germany. The top institutions in terms of the number of published articles are the Norwegian University of Science and Technology in the Kingdom of Norway, followed by the Central South University in China. Tuomi has shown significant long-term publication impact, while Zhou ranks among the most frequently cited authors. *Diabetes Care* is one of the most important scientific journals in diabetology with the highest impact factor of 16.2. This abstract summarizes a comprehensive bibliometric analysis that provides insights into the global research field of LADA type, underscoring the importance of international collaboration and the significant contributions of leading countries and institutions in shaping our understanding of this complex subtype of diabetes.

## 1. Introduction

Type 2 diabetes (T2D) mellitus (T2DM) is one of the global health challenges of the 21st century. The prevalence of T2DM has risen dramatically in recent decades. According to the World Health Organization (WHO), approximately 422 million people worldwide currently have diabetes [1]. The IDF Diabetes Atlas reports that 537 million people had diabetes in 2021, with projections indicating this number will reach 643 million by 2030 and 783 million by 2045 [2]. The majority of individuals with diabetes reside in low- and middle-income countries, and 1.5 million deaths annually are directly attributed to the disease [1].

Traditionally, researchers have presented T2DM as a homogeneous disease; however, an increasing number of evidence suggests its heterogeneity [3]. Research conducted in various countries has confirmed that T2DM consists of a complex array of subtypes. Each subtype has unique genetic, clinical, and metabolic features [4–7]. This diversity among T2DM subtypes suggests that patients can significantly differ in glycemic levels, degree of insulin resistance, duration of the disease, presence of complications, genetic predisposition, lifestyle, and personal treatment preferences. One of the subtypes identified within this spectrum is latent autoimmune diabetes in adults (LADA), often referred to as “Type 1.5 diabetes.”

In 2019, the WHO classified LADA type diabetes as a form of diabetes that combines characteristics of both T1DM and T2DM [8], in which markers of autoimmune damage to islet cells are found [9]. Experts categorize LADA as a form of T1D characterized by a slow progression of autoimmune diabetes, and it represents a significant proportion of T1D cases in adults. Clinically, however, it manifests as T2D, being insulin-independent at the time of disease onset, with clinical manifestations including various degrees of beta-cell autoimmunity, insulin resistance, and a wide range of genetic predispositions [10–12]. It differs from classical T1DM and T2DM in genetics, autoimmune response, rate of islet function decline, and clinical metabolic characteristics [13]. The proportion of LADA in T2DM cases is approximately 10% [14], making it the most frequent form of diabetes in adults, exceeding the prevalence of adult-onset T1DM by a factor of 3.3–12.2 [15]. This subtype plays a crucial role in the clinical management, diagnosis, and treatment of diabetes, reflecting its significant role in understanding and addressing diabetes more effectively [13, 16]. Experts also refer to LADA as NIRAD (noninsulin-requiring autoimmune diabetes) [17], AIDA (autoimmune diabetes in adults), and SPIDDM (slow progressing insulin-dependent diabetes mellitus) [18].

The bibliometric analysis is an important tool in assessing research activity and research trends on a particular topic for future planning and funding. This integrated approach allows the identification of emerging trends and main aspects of the disease. Researchers have conducted previous bibliometric analyses on diabetes mellitus, including diabetic nephropathy and retinopathy [19], COVID-19 interactions [20], gestational diabetes in Southeast Asia [21], diabetes nursing research trends [22], global diabetes management [23], diabetes research in Nigeria [24], gut microbiota links [25], periodontitis [26], and pressure ulcers in diabetic patients [27]. However, in the available databases, we did not find any bibliometric analysis related to LADA type diabetes. The aim of this study is to analyze the main research field on LADA type, including analysis of countries, institutions, journals, authors, and keywords. This analysis includes a wide range of analytical activities, from reviewing recent publications to analyzing collaborative networks and highlighting relevant topics.

## 2. Materials and Methods

### 2.1. Search Strategy

**Figure 1:**
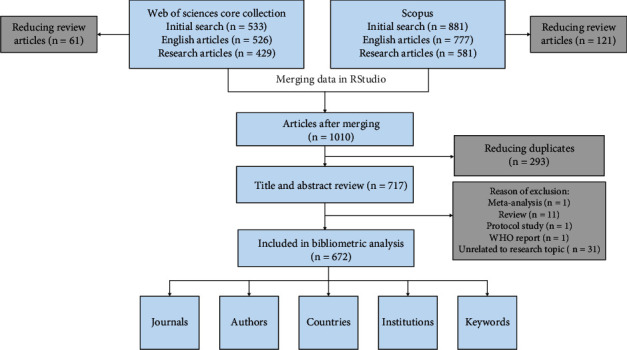
Search process.

This research utilized a descriptive bibliometric design. We collected and analyzed data from 672 publications indexed in the Web of Science and Scopus databases, covering the period from 1994 to January 2024. The bibliometric analysis included English-language research articles that involved studies on patients with LADA type diabetes, aged 18 years or older. Articles that were not original research or not relevant to the study topic were excluded from the analysis. All publications were exported in plain text format for Wos-CC and BibTeX format for Scopus, which contains bibliographic information, keyword information, citation information, etc. Both datasets were merged using a code in RStudio, and the combined data was exported in xlsx format. The detailed search process is illustrated in Figure 1. Tables S1, S2, and S3 present the RStudio merging code and search formula. The content analysis included the annual distribution of the number of target documents; the countries, institutions, and journals that published these articles; authors; keywords used in the articles; and changes in keyword frequency over time.

### 2.2. Study Selection and Data Extraction

We applied a two-stage process to select articles according to the inclusion criteria. In the first stage, two authors (B.T. and K.K.) independently evaluated the titles and abstracts of articles identified through the search strategy. Articles approved by both authors were analyzed full text. In case of disagreement, a third author (Y.B.) made the final decision.

### 2.3. Performance Analysis

We carried out the performance analysis and generated science maps with the help of the Bibliometrix R package (http://www.bibliometrix.org) within the RStudio v.4.3.2 (Posit Software, PBC Boston, Massachusetts, United States). Data analysis was carried out with the help of the Biblioshiny application. The annual scientific production and average citations per year plots generated by Biblioshiny were subsequently recreated in Flourish.studio (https://flourish.studio/).

## 3. Results

### 3.1. Summary of the Papers

Our study analyzed 672 documents from 261 sources, over the period of 1994–2024, including 7562 references and identifying 1086 unique author keywords. Approximately 20% of the authors participated in collaborative studies.

### 3.2. Annual Analysis of Publications

**Figure 2:**
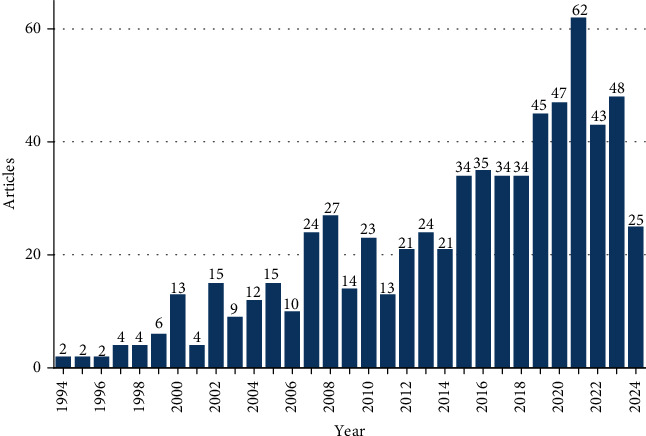
Annual publication trends over time (1994–2024).

The publications have shown a gradual increase from 1994 to 2024. From 2015 to 2018, there was a stable number of publications, ranging between 34 and 35 articles per year. From 2019 to 2020, there was a notable increase in the number of articles, with approximately 10 more publications compared to the previous years. Particularly high rates were observed in 2021, with 62 articles published. However, after peaking in 2021, which marked the highest number of publications, there was a decline of around 20 articles from 2022 to 2023 (Figure 2).

### 3.3. Countries and Affiliations

**Figure 3:**
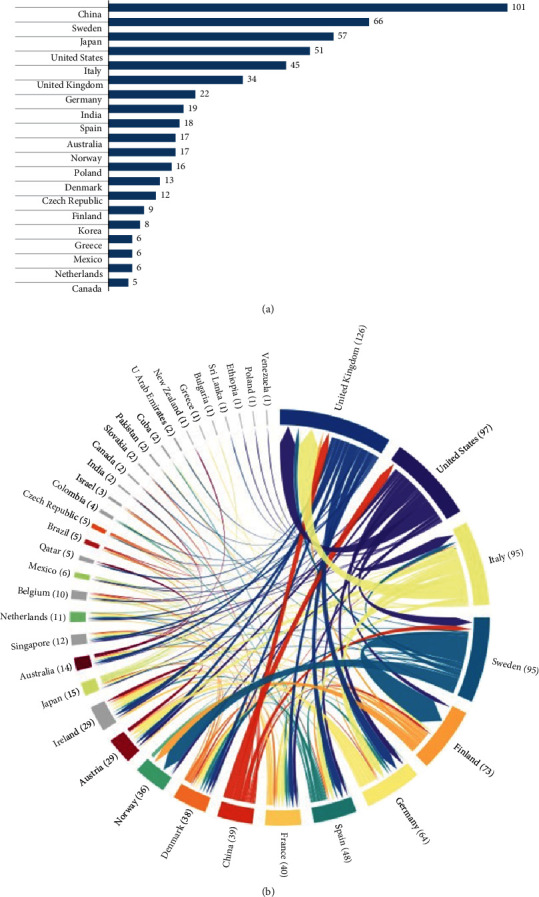
Top 20 countries with the highest number of articles by corresponding authors (a). Global research collaboration network (b).

Figure 3(a) shows the top 20 countries by number of articles produced. China tops the list with 101 articles, forming 14.9% of the total number of articles. However, the United Kingdom stands out as the most active country with 126 collaborations, especially with 64 joint projects with Germany (Figure 3(b)). Additionally, the United States, Italy, and Sweden follow with approximately 95 collaborations, respectively, emphasizing their significant role in the global partnership. Finland emerges as the main recipients of collaborations, with 33 projects, respectively, emphasizing the importance of connection with Sweden. Furthermore, Sweden and Italy have 2274 and 1779 of total citations, respectively, while Finland and Australia demonstrate higher citation rates per article 48.30 and 45.90, indicating impactful research contributions to the field of LADA type (Table 1). The top institutions in terms of the number of published articles are the Norwegian University of Science and Technology in the Kingdom of Norway with 111 articles, followed by the Central South University in China with 106 articles, the University of Helsinki in Finland with 71 articles, and Swedish institutions such as Karolinska Institutet, Lund University, and Uppsala University that stand out prominently with a total of 104 articles. This contribution to the scientific community emphasizes Sweden's strong academic position, which is reflected in the impressive total number of citations of Swedish papers (Table 2).

### 3.4. The Number of Publications and Citations by Different Authors

**Figure 4:**
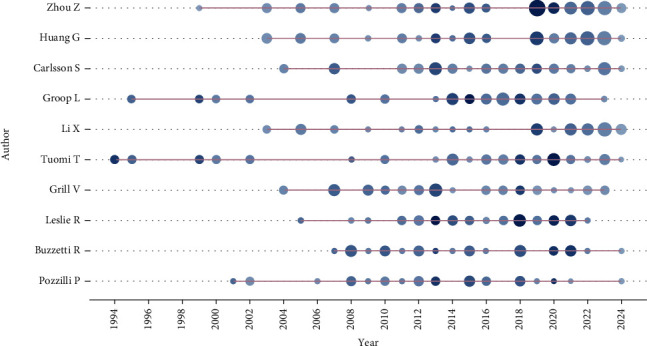
Authors' production over time.

Tuomi demonstrates longitudinal citation impact from 1994 to 2024, with the most cited period being in 2020, averaging 34,400 average annual citations. Considerable, in 1994, Tuomi collaborated with Professor Zimmet, and their work remains the most cited article to date. Furthermore, Author Groop has shown high scholarly output and citation impact, with the highest average annual citations in 2015, reaching 33,600 citations per year (Figure 4). The first two places in top 10 top cited authors were occupied by researchers from Central South University in Changsha, China: Zhou, Zhiguang, and Huang, Gan, with 60 and 50 articles, respectively. However, Leslie David and Pozzilli published 33 and 29 articles with the highest *H*-index 64 and 68, respectively (Table 3).

### 3.5. Journals

**Figure 5:**
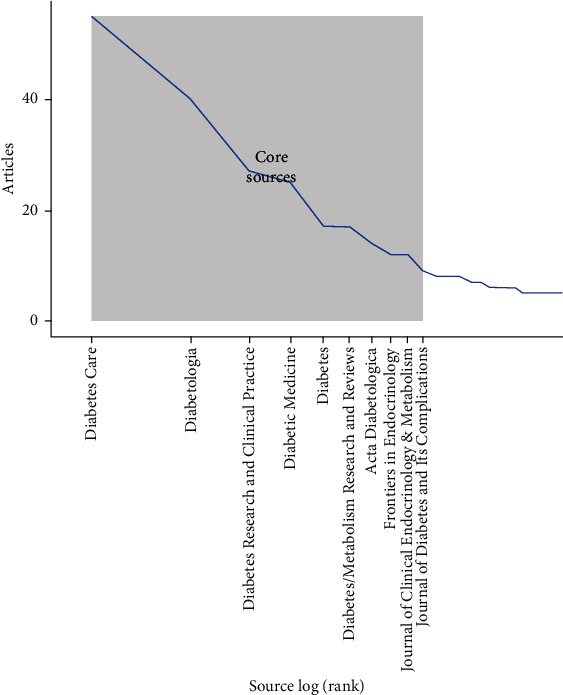
Core sources by Bradford's law.

Bradford's law concept of core sources is based on the idea that a small number of journals produce the vast majority of articles in a particular field [28].  Figure 5 shows the top 11 journals that are considered primary in the field of LADA type diabetes. The distribution of articles follows Bradford's law, with journals like *Diabetes Care* with 55 articles, *Diabetologia* with 40 articles, and *Diabetes Research and Clinical Practice* with 27 articles forming Zone 1, the core group that contributes a significant share of the research in the field. This pattern of publication frequency demonstrates an inverse relationship similar to Zipf's law, where the most productive journals significantly outnumber lower-ranked ones, reinforcing the core–periphery model of information dissemination. A significant drop in the number of publications is observed after the first three journals (*Diabetes Care*, *Diabetologia*, *and Diabetes Research and Clinical Practice*), which aligns with Bradford's law. Additionally, the journal *Diabetes Care* has the highest impact factor (IF) of 16.2. Moreover, the journals *Diabetologia* and *Diabetes/Metabolism Research and Reviews* have IFs of 8.2 and 8.0, respectively. It is noteworthy that 50% of all the journals rank in the top quartile (Q1) of the Endocrinology & Metabolism category in the Science Citation Index Expanded (SCIE) (Table 4).

### 3.6. Most Relevant Documents and Trend Topics

**Figure 6:**
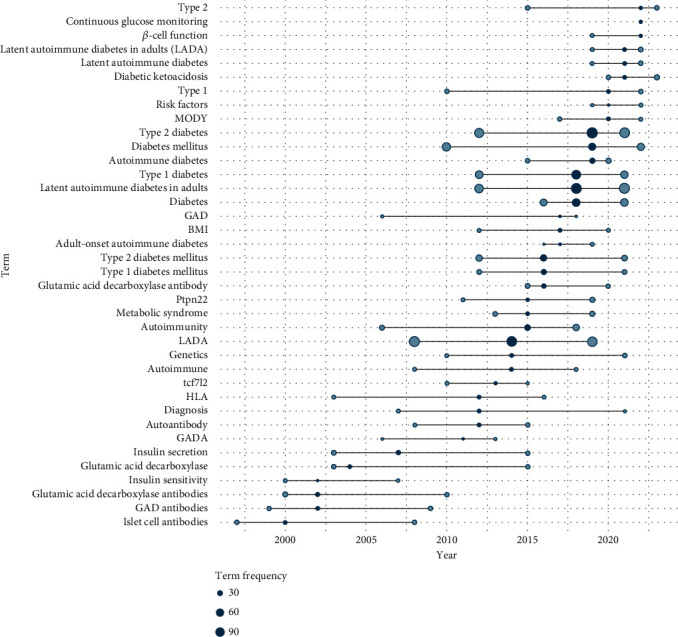
Trend topics.

The author keyword *type 2 diabetes* is the most frequently used in the investigated field, with a frequency of 115 occurrences, reaching its peak usage in 2019. Additionally, the keyword *latent autoimmune diabetes in adults* was used 112 times, marking its highest frequency in 2018. Keywords such as *islet cell antibodies* and *gad antibodies* were prominent in the late 1990s and early 2000s, with a frequency of 11 and 6, respectively. Recent years have highlighted *type 1 diabetes mellitus* with a frequency of 81 times as key research topics. Notably, certain keywords such as *autoimmunity* and *gad* frequently appeared together, indicating collaborative studies or related research themes (Figure 6). The top three most cited articles on LADA type diabetes, as listed in our bibliometric analysis, have significantly the understanding of this condition. In instance, Zimmet et al. in 1994 highlight the role of antibodies to glutamate decarboxylase in the diagnosis and prediction of insulin dependency in LADA type with 398 citations [29]. The results of Tuomi et al. with 390 citations in 1999 showed that patients with positive GAD antibodies differ from those with negative antibodies in *β*-cell function in both T1D and T2D [30]. They proposed to define LADA as the presence of GAD antibodies in patients over 35 years old at the onset of T2D. Prasad and Groop [31] highlight the impact of the technical revolution in genetics on identifying numerous genetic variants associated with T2D, published in 2019 with 322 citations (Table 5).

## 4. Discussion

We analyzed 672 publications on LADA type diabetes research from 1994 to 2024. The steady increase in the number of articles highlights the sustained interest in this research topic. The obtained findings include publication metrics, trends in scientific output, countries and their collaboration patterns, contributions from institutions, journal preferences, author productivity, and the topic trends indicated by author keywords within this field.

The analysis revealed a significant increase in the number of research articles in LADA type diabetes in recent years. This upward trend indicates a growing recognition of the critical importance of understanding the main cause and pathogenesis of LADA type diabetes mellitus. The increasing volume of scientific research underscores the urgent need to address the challenges associated with diagnostic accuracy, treatment efficacy, and patient management in LADA type diabetes. The solution to these problems involves the technical revolution in genetics to identify numerous genetic variants associated with T2D [31]. Zimmet et al. highlight the role of antibodies to glutamate decarboxylase in the diagnosis and prediction of insulin dependency in LADA type [29].

China emerges as the primary leading countries in this research field, exhibiting substantial levels of publications. International scientific collaboration, notably involving the United Kingdom, United States, Italy, and Sweden, plays a pivotal role in advancing science and technology in this domain. Countries demonstrating active engagement in this field typically correspond to those characterized by high-income levels. This trend implies that developed economies, endowed with significant financial and technological resources, possess the capability to conduct research within this domain. Our investigation has elucidated a notable absence of contributions from low-income countries among the authors, indicating that these regions encounter more conspicuous and formidable barriers due to constraints in research funding, inadequate institutional support, and restricted educational opportunities prevalent in low-income settings [39]. Additionally, language barriers and constrained access to technical resources impede active engagement in research endeavors.

The journals included in the core sources in the field of LADA encompass studies specializing in endocrinology and metabolism. Moreover, core sources comprise journals characterized by high IFs, signifying their considerable influence. The curation of authoritative peer-reviewed publications is essential for upholding trust in research outcomes, thereby safeguarding the integrity of the presented data. This is especially critical given the reliance of policymakers and healthcare practitioners on robust evidence for informed decision-making [30].

Over the past decades, key studies have significantly advanced the understanding of LADA type diabetes. There is a trend toward increasing knowledge of the mechanisms of development and specific biomarkers of LADA type, which provides prospects for refining diagnosis and stratifying therapy. The work of Zimmet et al. highlighted the role of antibodies to glutamate decarboxylase in the diagnosis and prediction of insulin dependence in LADA type [29]. Tuomi et al.'s study has led to a better understanding of the pathophysiology of LADA and the differences between types of diabetes [30]. Prasad and Groop highlight the importance of the technological revolution in genetics. However, despite these advances, the genetic landscape of T2D remains incomplete and explains only a small portion of the disease's heritability [31]. Pozzilli and Di Mario highlighted the critical importance of diagnosing LADA patients, therapeutic interventions, and protection of residual C-peptide secretion [40].

Tracking keyword trends demonstrates how the focus of diabetes research has shifted and expanded over time. The discussion centered around *type 1 diabetes* and *type 2 diabetes*, with emphasis on *cell antibodies* and *gad antibodies*. This focus broadened to include autoimmune mechanisms within *type 1 diabetes* and *type 2 diabetes mellitus* and eventually spotlighted LADA type as its own entity. The peak in LADA type mentions in 2010 suggests a notable increase in research interest and potential breakthroughs. In 2018, the attention pivoted back to the broader term *latent autoimmune diabetes in adults*, reflecting a rejuvenated research interest in the adult-onset form of the disease. These changes in the research landscape echo a growing acknowledgement of LADA type significance in diabetes studies.

## 5. Conclusion

This bibliometric review has underscored the necessity for continued research into LADA type, particularly to address gaps in diagnosis and treatment strategies, which are often complicated by its similarities and frequent confusion with T2DM. Future research should also be aimed at fostering global cooperation and resource sharing to mitigate the disparity in research capabilities between developed and developing countries, ensuring a more equitable distribution of knowledge and advancements in diabetes care.

## Figures and Tables

**Table 1:** Most cited countries.

**Rank**	**Country**	**Total citations**	**Average article citations**
1	Sweden	2274	34.50
2	Italy	1779	39.50
3	China	1680	16.60
4	United Kingdom	1176	34.60
5	United States	1115	21.90
6	Japan	1017	17.80
7	Australia	781	45.90
8	Norway	526	30.90
9	Germany	491	22.30
10	Finland	435	48.30

**Table 2:** Most relevant affiliations.

**Rank**	**Affiliation**	**Articles**	**Country**
1	Norwegian University of Science and Technology	111	Kingdom of Norway
2	Central South University	106	China
3	University of Helsinki	71	Finland
4	Karolinska Institutet	33	Sweden
5	Lund University	46	Sweden
6	Helsinki University Central Hospital	37	Denmark
7	University of London	29	United Kingdom
8	Queen Mary University London	29	United Kingdom
9	Uppsala University	25	Sweden
10	University of Pennsylvania	23	United States

**Table 3:** Most relevant cited authors.

**Rank**	**Authors**	**Articles**	**H** **-index**	**Affiliation**
1	Zhou, Zhiguang	60	54	Central South University, Changsha, China
2	Huang, Gan	50	33	Central South University, Changsha, China
3	Groop, Leif C.	44	51	Lunds Universitet, Lund, Sweden
4	Carlsson, Sofia	44	30	Karolinska Institutet, Stockholm, Sweden
5	Li, Xia	43	33	Central South University, Changsha, China
6	Tuomi, Tiinamaija	42	64	Helsinki University Hospital, Helsinki, Finland
7	Grill, Valdermar E. R.	37	55	Norges Teknisk-Naturvitenskapelige Universitet, Trondheim, Norway
8	Leslie, David R. G.	33	64	Queen Mary University of London, London, United Kingdom
9	Buzzetti, Raffaella	30	53	Sapienza Università di Roma, Italy
10	Pozzilli, Paolo	29	68	Barts and The London School of Medicine and Dentistry, London, United Kingdom

**Table 4:** Most relevant sources.

**Rank**	**Sources name**	**Articles**	**IF**	**JCR category (quartile)**
1	*Diabetes Care*	55	16.2	Endocrinology & Metabolism—SCIE (Q1)
2	*Diabetologia*	40	8.2	Endocrinology & Metabolism—SCIE (Q1)
3	*Diabetes Research and Clinical Practice*	27	5.1	Endocrinology & Metabolism—SCIE (Q2)
4	*Diabetic Medicine*	25	3.5	Endocrinology & Metabolism—SCIE (Q3)
5	*Diabetes*	17	7.7	Endocrinology & Metabolism—SCIE (Q1)
6	*Diabetes/Metabolism Research and Reviews*	17	8.0	Endocrinology & Metabolism—SCIE (Q1)
7	*Acta Diabetologica*	14	3.8	Endocrinology & Metabolism—SCIE (Q2)
8	*Journal of Clinical Endocrinology & Metabolism*	12	5.8	Endocrinology & Metabolism—SCIE (Q1)
9	*Frontiers in Endocrinology*	12	5.2	Endocrinology & Metabolism—SCIE (Q1)
10	*Journal of Diabetes and Its Complications*	9	3.0	Endocrinology & Metabolism—SCIE (2)

**Table 5:** The 10 most frequently cited quality papers focusing on LADA type.

**Rank**	**Article**	**1** ^**st**^ ** author**	**DOI**	**Total citations**	**TC per year**	**Year of publication**
1	Latent Autoimmune Diabetes Mellitus in Adults (LADA): the Role of Antibodies to Glutamic Acid Decarboxylase in Diagnosis and Prediction of Insulin Dependency [29]	P. Zimmet	10.1111/j.1464-5491.1994.tb00275.x	398	12.84	1994
2	Clinical and Genetic Characteristics of Type 2 Diabetes With and Without GAD Antibodies [30]	T. Tuomi	10.2337/diabetes.48.1.150	390	15.00	1999
3	Genetics of Type 2 Diabetes—Pitfalls and Possibilities [31]	R. B. Prasad	10.3390/genes6010087	322	32.20	2019
4	Autoimmune Diabetes Not Requiring Insulin at Diagnosis (Latent Autoimmune Diabetes of the Adult): Definition, Characterization, and Potential Prevention [32]	P. Pozzilli	10.2337/diacare.24.8.1460	240	10.0	2001
5	Adult-Onset Autoimmune Diabetes in Europe Is Prevalent With a Broad Clinical Phenotype [33]	M. I. Hawa	10.2337/dc12-0931	233	19.42	2009
6	Frequency, Immunogenetics, and Clinical Characteristics of Latent Autoimmune Diabetes in China (LADA China Study) [34]	Z. Zhou	10.2337/db12-0207	199	16.58	2013
7	Clinical Evidence for the Safety of GAD65 Immunomodulation in Adult-Onset Autoimmune Diabetes [35]	C. D. Agardh	10.1186/s12889-015-1591-y	187	9.35	2005
8	High Titer of Autoantibodies to GAD Identifies a Specific Phenotype of Adult-Onset Autoimmune Diabetes [36]	R. Buzzetti	10.2337/dc06-1696	180	10.00	2007
9	GAD Antibodies in NIDDM: Ten-Year Follow-Up From the Diagnosis [37]	L. K. Niskanen	10.2337/diacare.18.12.1557	171	5.70	1995
10	Genetic Similarities Between Latent Autoimmune Diabetes in Adults, Type 1 Diabetes, and Type 2 Diabetes [38]	C. Cervin	10.2337/db07-0299	166	9.76	2008

## Data Availability

The data that support the findings of this study are available from the corresponding author upon reasonable request.

## Supporting Information

Supporting Information Additional supporting information can be found online in the Supporting Information section. Tables S1 and S2 provide the search queries used for retrieving relevant articles from the Scopus and Web of Science databases, respectively. Table S3 presents the R code used for merging data from the Web of Science and Scopus databases. The code imports datasets from both sources, combines them, removes duplicates, and exports the merged data into an Excel file.
