# Supplementary material for: Global Trends in LADA Type Diabetes Research: A Bibliometric Analysis of Publications from Web of Science and Scopus, 1994–2024
Source: J Diabetes Res. 2024 Oct 14;2024:4960075. doi: 10.1155/2024/4960075 (PMC11493478; doi:10.1155/2024/4960075)
Supplement: Supporting Information — Additional supporting information can be found online in the Supporting Information section. Tables S1 and S2 provide the search queries used for retrieving relevant articles from the Scopus and Web of Science databases, respectively. Table S3 presents the R code used for merging data from the Web of Science and Scopus databases. The code imports datasets from both sources, combines them, removes duplicates, and exports the merged data into an Excel file. [file 4960075.f1.doc]

**Global trends of LADA type diabetes research: A bibliometric analysis of publications from 1994 to 2024**

**Khatimya Kudabayeva^1^, Bibigul Tleumagamabetova^2,*^, Yerlan Bazargaliyev^1^, Raikul Kosmuratova^1^, Aliya Zhylkybekova^3^**

^1^ Department of Internal Diseases № 1, West Kazakhstan Marat Ospanov Medical University, Aktobe, Kazakhstan. [hatimakudabayeva@gmail.com](mailto:hatimakudabayeva@gmail.com) (Kh.K); [Yerlanbazargaliyev@gmail.com](mailto:Yerlanbazargaliyev@gmail.com) (Ye.B); [kosmuratova.raikul@mail.ru](mailto:kosmuratova.raikul@mail.ru) (R.K.)

^2^ Department of Propaedeutics of Internal Disease, West Kazakhstan Marat Ospanov Medical University, Aktobe, Kazakhstan. [tleumagambetova@zkmu.kz](mailto:tleumagambetova@zkmu.kz)

^3^ Department of Evidence-Based Medicine and Scientific Management, West Kazakhstan Marat Ospanov Medical University, 030012 Aktobe, Kazakhstan. zhylkybekovaa@gmail.com

*Corresponding author: +77078115119

[tleumagambetova@zkmu.kz](mailto:tleumagambetova@zkmu.kz)

Table S1. Query for Scopus

| Query |
| --- |
| ( TITLE-ABS-KEY “LADA” OR “Diabetes Mellitus Type 1.5” OR “Type 1.5 Diabetes Mellitus” OR “Type 1.5 Diabetes” OR “Diabetes, Type 1.5” OR “LADA, Latent Autoimmune Diabetes in Adults” OR “Latent Autoimmune Diabetes of Adults” OR “Non Insulin Requiring Autoimmune Diabetes” OR “Autoimmune Diabetes in Adults” OR “Slow Progressing Insulin Dependent Diabetes Mellitus” OR “NIRAD” OR “AIDA” OR “SPIDDM”) AND “Diabetes Mellitus, Type 2” OR “Diabetes Mellitus, Noninsulin-Dependent” OR “Diabetes Mellitus, Ketosis-Resistant” OR “Diabetes Mellitus, Ketosis Resistant” OR “Ketosis-Resistant Diabetes Mellitus” OR “Diabetes Mellitus, Non Insulin Dependent” OR “Diabetes Mellitus, Non-Insulin-Dependent” OR “Non-Insulin-Dependent Diabetes Mellitus” OR “Diabetes Mellitus, Stable” OR “Stable Diabetes Mellitus” OR “Diabetes Mellitus, Type II” OR “NIDDM” OR “Diabetes Mellitus, Noninsulin Dependent|” OR “Diabetes Mellitus, Maturity-Onset” OR “Diabetes Mellitus, Maturity Onset” OR “Maturity-Onset Diabetes Mellitus” OR “Maturity Onset Diabetes Mellitus” OR “MODY” OR “Diabetes Mellitus, Slow-Onset” OR “Diabetes Mellitus, Slow Onset” OR “Slow-Onset Diabetes Mellitus” OR “Type 2 Diabetes Mellitus” OR “Noninsulin-Dependent Diabetes Mellitus” OR “Noninsulin Dependent Diabetes Mellitus” OR “Maturity-Onset Diabetes” OR “Diabetes, Maturity-Onset” OR “Maturity Onset Diabetes” OR “Type 2 Diabetes” OR “Diabetes, Type 2” OR “Diabetes Mellitus, Adult-Onset” OR “Adult-Onset Diabetes Mellitus” OR “Diabetes Mellitus, Adult Onset” AND ( LIMIT-TO ( LANGUAGE , "English" ) ) |

Table S2. Query for Web of Science database

| № | Queries |
| --- | --- |
| #1 | “LADA” OR “Diabetes Mellitus Type 1.5” OR “Type 1.5 Diabetes Mellitus” OR “Type 1.5 Diabetes” OR “Diabetes, Type 1.5” OR “LADA, Latent Autoimmune Diabetes in Adults” OR “Latent Autoimmune Diabetes of Adults” OR “Non Insulin Requiring Autoimmune Diabetes” OR “AutoImmune Diabetes in Adults” OR “Slow Progressing Insulin Dependent Diabetes Mellitus” OR “NIRAD” OR “AIDA” OR “SPIDDM” (Title) or “LADA” OR “Diabetes Mellitus Type 1.5” OR “Type 1.5 Diabetes Mellitus” OR “Type 1.5 Diabetes” OR “Diabetes, Type 1.5” OR “LADA, Latent Autoimmune Diabetes in Adults” OR “Latent Autoimmune Diabetes of Adults” OR “Non Insulin Requiring Autoimmune Diabetes” OR “AutoImmune Diabetes in Adults” OR “Slow Progressing Insulin Dependent Diabetes Mellitus” OR “NIRAD” OR “AIDA” OR “SPIDDM” (Author Keywords) or “LADA” OR “Diabetes Mellitus Type 1.5” OR “Type 1.5 Diabetes Mellitus” OR “Type 1.5 Diabetes” OR “Diabetes, Type 1.5” OR “LADA, Latent Autoimmune Diabetes in Adults” OR “Latent Autoimmune Diabetes of Adults” OR “Non Insulin Requiring Autoimmune Diabetes” OR “AutoImmune Diabetes in Adults” OR “Slow Progressing Insulin Dependent Diabetes Mellitus” OR “NIRAD” OR “AIDA” OR “SPIDDM” (Abstract) |
| #2 | “Diabetes Mellitus, Type 2” OR “Diabetes Mellitus, Noninsulin-Dependent” OR “Diabetes Mellitus, Ketosis-Resistant” OR “Diabetes Mellitus, Ketosis Resistant” OR “Ketosis-Resistant Diabetes Mellitus” OR “Diabetes Mellitus, Non Insulin Dependent” OR “Diabetes Mellitus, Non-Insulin-Dependent” OR “Non-Insulin-Dependent Diabetes Mellitus” OR “Diabetes Mellitus, Stable” OR “Stable Diabetes Mellitus” OR “Diabetes Mellitus, Type II” OR “NIDDM” OR “Diabetes Mellitus, Noninsulin Dependent|” OR “Diabetes Mellitus, Maturity-Onset” OR “Diabetes Mellitus, Maturity Onset” OR “Maturity-Onset Diabetes Mellitus” OR “Maturity Onset Diabetes Mellitus” OR “MODY” OR “Diabetes Mellitus, Slow-Onset” OR “Diabetes Mellitus, Slow Onset” OR “Slow-Onset Diabetes Mellitus” OR “Type 2 Diabetes Mellitus” OR “Noninsulin-Dependent Diabetes Mellitus” OR “Noninsulin Dependent Diabetes Mellitus” OR “Maturity-Onset Diabetes” OR “Diabetes, Maturity-Onset” OR “Maturity Onset Diabetes” OR “Type 2 Diabetes” OR “Diabetes, Type 2” OR “Diabetes Mellitus, Adult-Onset” OR “Adult-Onset Diabetes Mellitus” OR “Diabetes Mellitus, Adult Onset” (Title) or “Diabetes Mellitus, Type 2” OR “Diabetes Mellitus, Noninsulin-Dependent” OR “Diabetes Mellitus, Ketosis-Resistant” OR “Diabetes Mellitus, Ketosis Resistant” OR “Ketosis-Resistant Diabetes Mellitus” OR “Diabetes Mellitus, Non Insulin Dependent” OR “Diabetes Mellitus, Non-Insulin-Dependent” OR “Non-Insulin-Dependent Diabetes Mellitus” OR “Diabetes Mellitus, Stable” OR “Stable Diabetes Mellitus” OR “Diabetes Mellitus, Type II” OR “NIDDM” OR “Diabetes Mellitus, Noninsulin Dependent|” OR “Diabetes Mellitus, Maturity-Onset” OR “Diabetes Mellitus, Maturity Onset” OR “Maturity-Onset Diabetes Mellitus” OR “Maturity Onset Diabetes Mellitus” OR “MODY” OR “Diabetes Mellitus, Slow-Onset” OR “Diabetes Mellitus, Slow Onset” OR “Slow-Onset Diabetes Mellitus” OR “Type 2 Diabetes Mellitus” OR “Noninsulin-Dependent Diabetes Mellitus” OR “Noninsulin Dependent Diabetes Mellitus” OR “Maturity-Onset Diabetes” OR “Diabetes, Maturity-Onset” OR “Maturity Onset Diabetes” OR “Type 2 Diabetes” OR “Diabetes, Type 2” OR “Diabetes Mellitus, Adult-Onset” OR “Adult-Onset Diabetes Mellitus” OR “Diabetes Mellitus, Adult Onset” (Author Keywords) or “Diabetes Mellitus, Type 2” OR “Diabetes Mellitus, Noninsulin-Dependent” OR “Diabetes Mellitus, Ketosis-Resistant” OR “Diabetes Mellitus, Ketosis Resistant” OR “Ketosis-Resistant Diabetes Mellitus” OR “Diabetes Mellitus, Non Insulin Dependent” OR “Diabetes Mellitus, Non-Insulin-Dependent” OR “Non-Insulin-Dependent Diabetes Mellitus” OR “Diabetes Mellitus, Stable” OR “Stable Diabetes Mellitus” OR “Diabetes Mellitus, Type II” OR “NIDDM” OR “Diabetes Mellitus, Noninsulin Dependent|” OR “Diabetes Mellitus, Maturity-Onset” OR “Diabetes Mellitus, Maturity Onset” OR “Maturity-Onset Diabetes Mellitus” OR “Maturity Onset Diabetes Mellitus” OR “MODY” OR “Diabetes Mellitus, Slow-Onset” OR “Diabetes Mellitus, Slow Onset” OR “Slow-Onset Diabetes Mellitus” OR “Type 2 Diabetes Mellitus” OR “Noninsulin-Dependent Diabetes Mellitus” OR “Noninsulin Dependent Diabetes Mellitus” OR “Maturity-Onset Diabetes” OR “Diabetes, Maturity-Onset” OR “Maturity Onset Diabetes” OR “Type 2 Diabetes” OR “Diabetes, Type 2” OR “Diabetes Mellitus, Adult-Onset” OR “Adult-Onset Diabetes Mellitus” OR “Diabetes Mellitus, Adult Onset” (Abstract) |
| #3 | #1 and #2 |

Table S3. R-code for merging data

| Code |
| --- |
| library(bibliometrix)  library(openxlsx)  ## importing web of science dataset  web_data<-convert2df("abs.txt")  ## importing scopus dataset  scopus_data<-convert2df("abs.bib",dbsource="scopus",format="bibtex")  ##combined both datasets  combined<-mergeDbSources(web_data,scopus_data,remove.duplicated=T)  ##exporting file  write.xlsx(combined,"combinedabs.xlsx") |
